# New Insights into Antiviral Natural Formulations: Biopolymeric Films for the Prevention and Treatment of a Wide Gamma of Viral Infections

**DOI:** 10.3390/v17020216

**Published:** 2025-02-01

**Authors:** Victoria Belén Ayala-Peña, Ana Karen Jaimes, Ana Lucía Conesa, Cybele Carina García, Claudia Soledad Sepulveda, Fernando Gaspar Dellatorre, Ezequiel Latour, Nora Marta Andrea Ponce, Vera Alejandra Álvarez, Verónica Leticia Lassalle

**Affiliations:** 1 Departamento de Biología, Bioquímica y Farmacia, Universidad Nacional del Sur, San Juan 670, Bahía Blanca 8000, Argentina; ana.conesa.97@gmail.com; 2 Consejo Nacional de Investigaciones Científicas y Técnicas (CONICET), Buenos Aires 8000, Argentina; anak.jaimes.97@gmail.com (A.K.J.); cybele.garcia@gmail.com (C.C.G.); claudia@qb.fcen.uba.ar (C.S.S.); dellatorcnp@gmail.com (F.G.D.); aponce@qo.fcen.uba.ar (N.M.A.P.); alvarezvera@gmail.com (V.A.Á.); 3 INQUISUR, Departamento de Química, Universidad Nacional del Sur, Av. Alem 1253, Bahía Blanca 8000, Argentina; 4 Facultad de Ingeniería, INTEMA, Universidad Nacional de Mar del Plata (UNMdP), Av. Colón 10850, Mar del Plata 2695, Argentina; 5 Departamento de Química Biológica, Facultad de Ciencias Exactas y Naturales, Universidad de Buenos Aires, Av. Int. Güiraldes 2610, Buenos Aires 1053, Argentina; 6 Facultad Regional Chubut, Grupo de Investigación y Desarrollo Tecnológico en Acuicultura y Pesca (GIDTAP), Universidad Tecnológica Nacional, Av. del Trabajo 1536, Puerto Madryn 3000, Argentina; ezequiellatour@gmail.com; 7 Departamento de Química Orgánica, Facultad de Ciencias Exactas y Naturales and Centro de Investigaciones en Hidratos de Carbono (CIHIDECAR-UBA), Universidad de Buenos Aires, Intendente Güiraldes 2160, Buenos Aires 1053, Argentina

**Keywords:** antiviral, fucoidan, chitosan, scavenger, formulations

## Abstract

Viral infections remain a major concern, as existing treatments often yield inadequate responses or lead to the development of antiviral resistance in some cases. Fucoidan extracted from *Undaria pinnatifida* (F) is a natural sulphated polysaccharide that exhibits antiviral action. Despite its potential, the biomedical application of F is limited due to its difficult administration through trans-mucosal, skin, or oral ingestion. The most effective way to solve these problems is to propose novel methods of administration aiming to ensure better contact between the biopolymers and pathogens, leading to their inactivation. In this work, the synthesis of films based on chitosan (Ch)-coupled F is reported, aiming to generate a synergic effect between both biopolymers in terms of their antiviral and antioxidant capability. Biocomposites were prepared by a sonochemical method. They were characterized to infer structural properties, functionality, and possible F-Ch interactions by using Zeta potential, FTIR, and XRD techniques. The biocomposites showed excellent film-forming ability. They also exhibited improved antioxidant activity with respect to F and Ch individually and proved to be non-cytotoxic. These results demonstrate, for the first time, the antiviral activity of F:Ch biocomposites against bovine coronavirus and human viruses (adenovirus, poliovirus, herpes simplex, and respiratory syncytial virus), which could be applied in film form to prevent or treat viral infections.

## 1. Introduction

Fucoidans are polysaccharides biosynthesized by brown algae that have attracted significant attention over the last decade due to their diverse biological properties. These polysaccharides contain large amounts of L-fucose and sulphate, together with minor proportions of other sugars like xylose, galactose, mannose, and uronic acids [1,2]. Extensive studies have highlighted their various effects, including anticoagulant, antitumor, anti-inflammatory, antithrombotic, antiviral, and antioxidant properties [3,4,5,6,7,8]. Such bioactivities have been attributed to some characteristics of fucoidan molecules, including their molecular weight, sulfation pattern, and sulfation degree [9]. Specifically, there are a large number of reports on the biological properties of fucoidans extracted from the seaweed *Undaria pinnatifida*, including antiviral activity [10] and antioxidant activity [5,11]. This species belongs to the family Alariaceae of the order Laminariales, which is native to the seas of China, Japan, and Korea and has been introduced to many other locations, including the Argentine Patagonian coasts. Despite the acknowledged antiviral properties of fucoidans, their bioavailability when administered orally is generally low [12,13]. Their antioxidant properties, however, make them very attractive in the cosmetic industry. Currently, fucoidans are proposed as the main component in certain creams due to their antioxidant properties, which prevent damage caused by free radicals generated during the aging process [14].

Chitosan is a natural biopolymer that can be obtained from the N-deacetylation of chitin, which is found, for example, in the exoskeletons of marine crustaceans and other biological systems such as fungi or bacteria [15]. The broad applicability of this biopolymer is extensive due to two main characteristics: its functionality (due to its amino and hydroxyl groups) and its safety. These features lead to interesting prospects for application in countless fields, with the biomedical field being one of the most explored [16]. Its mucoadhesive capacity, hemostatic effect, antibacterial effect, and biodegradability make it highly suitable for the design of drug systems. Studies have shown that chitosan possesses antiviral properties; however, it has been determined that these are limited [17,18,19]. It is thus tempting to propose that its use in combination with other compounds could enhance these effects, as the antiviral activity of chitosan has been shown to increase when it is combined with metal salts [20,21,22]. In this work, biocompatible formulations are presented. The combination of fucoidans and chitosan has not been sufficiently explored according to the existing literature. Background information is limited to a few studies involving the fabrication of fucoidan–chitosan nanomaterials with biomedical applications, such as nanoparticles for oral drug administration [23,24], hydrogels for skin regeneration in burn wounds [25], and antibacterial films [26], among other applications [27,28].

Viruses are non-living structures with genetic material encapsulated and enveloped or not by a membrane. Since viruses need a living cell to infiltrate and take over its metabolic system in order to receive energy and nutrients, they are unable to multiply and expand on their own. Many viruses, including respiratory viruses like adenovirus (ADV), respiratory syncytial virus (RSV), and severe acute respiratory syndrome coronavirus 2 (SARS-CoV-2), initially replicate at the mucosal level, posing further challenges. Since the COVID-19 pandemic, the SARS-CoV-2 virus has caused great loss, and the consequences of the pandemic are still being suffered. On the other hand, skin viral infections are highly prevalent worldwide, causing significant morbidity and potential complications. For example, Merkel cell carcinoma is an uncommon type of highly aggressive skin cancer [29]. Viral infection with Merkel cell polyomavirus and radiation exposure are the two leading factors implicated in its causation. This virus is a widespread pathogen that is not enveloped and has a DNA genome type. Beyond the initial viral rash, it can lead to the development of malignancies in some cases. Examples include lesions developed during Kaposi’s sarcoma, which is caused by herpes virus type 8, or mucocutaneous warts and cervical, anal, penile, and pharyngeal cancers associated with human papilloma virus (HPV). It is also important to highlight that even non-cancerous viruses pose significant risks, especially for immunocompromised individuals. Mother-to-child transmission of herpesvirus can cause severe consequences for the infant, including vision and hearing loss, as well as neurological problems. Herpes simplex type 1 (HSV-1) is one of the most prevalent viral pathogens in the adult population. While acyclovir is frequently used for treatment, it has limitations in certain age groups and can lead to resistance. Since viruses rely on the host cell for multiplication, creating safe and efficient antiviral drugs that do not interfere with host cell function is a significant challenge. Additionally, currently, available antiviral drugs are often limited, and all of them are specific to certain types of viruses. Therefore, the efficiency of many commercially available antiviral therapeutic agents is limited by the existence of unwanted side effects [30]. Furthermore, it has been demonstrated that the effectiveness of antiviral drugs and vaccines is significantly restricted by viral mutations [31]. Therefore, this study explores promising biopolymeric-based formulations combining broad-spectrum antiviral and antioxidant activity. These formulations have the potential to address any type of viral etiologic lesion, regardless of its viral structure (enveloped/non enveloped) or viral genome (DNA/RNA), allowing their application in a preventive or therapeutic capacity. Hence, in this work, we demonstrate, for the first time, their antiviral activity against enveloped viruses (including bovine coronavirus, which can be used as biosafe viral model of SARS-CoV 2) and non-enveloped viruses, as well as antioxidant properties of biomaterials based on fucoidan–chitosan.

## 2. Materials and Methods

### 2.1. Materials

Powdered chitosan (Ch) (apparent viscosity 16MPA.S; deacetylation degree 95.37%) was purchased from Farmacia Homeopática Pereda (Mar del Plata, Argentina). Fucoidan extract (F) was obtained from *Undaria pinnatifida* sporophylls collected in October 2015 in Golfo Nuevo, northern Patagonia, Argentina. The fucoidan extraction technique was performed as reported by Ponce et al. [32]; 10 g of dried and milled algae were extracted under magnetic stirring with 0.01 M HCl (pH = 2) at 25 °C for 6 h. Then, the mixture was centrifuged, and the extract (F) was neutralized, concentrated at reduced pressure, dialyzed (mol.wt cut off 6000–8000), and recovered by freeze-drying.

### 2.2. Preparation of Fucoidan–Chitosan (F:Ch) Biocomposites

Aqueous stock solutions of F of 1, 0.5 and 0.25% were prepared. A stock solution of Ch 3% in acetic acid (1%) was also prepared. Then, 100 mL of each F stock solution was combined with 16.66 mL of Ch stock solution to obtain the biomaterial’s precursors with F:Ch nominal mass ratios of 1:1, 1:2 and 2:1. The reaction mixtures were treated by ultrasound following the procedure reported by Barbosa et al. [23], with some modifications. An ultrasonic homogenizer pulse (DP0150-6 Benchmark Scientific, Sayreville, NJ, USA) with a 6 mm diameter titanium probe, 150 W, and 75% efficiency was used. Pulsed ultrasound irradiation (3s/7s) was employed with a certain power of amplitude for 30 s working with a frequency of 20 KHz. The temperature was controlled with a static water-cooling bath and maintained at room temperature (r.t.). Aqueous dispersions of biomaterials were obtained and stored at 4 °C. Fractions of them were used for the formation of film by casting in air at r.t.

### 2.3. Chemical Characterization

#### 2.3.1. Analytical Methods

Total carbohydrates were estimated by the phenol–H_2_SO_4_ method using fucose as standard [33], and the percentages of sulphate were measured by turbidimetry [34] after hydrolysis with 1 M HCl at 110 °C for 4.5 h.

Hydrolysis was carried out with 2 M CF_3_COOH (90 min, 120 °C) [35]. Hydrolyzates were derivatized to the aldononitrile acetates and analyzed by GLC using a capillary column (30 m × 0.25 mm) coated with SP-2330 (0.20 μm) on a Nexis 2030 gas chromatograph equipped with a flame ionization detector (FID). Nitrogen was used as the carrier gas, with a head pressure of 15 psi and a split ratio of 100:1. Chromatography runs were isothermal at 220 °C, while the injector and detector were set at 240 °C.

#### 2.3.2. X-Ray Diffraction

X-ray diffraction (XRD) data were collected using a PANalytical Empyrean 3 diffractometer with Ni-filtered CuKα radiation and a PIXcel3D detector. A voltage of 45 kV and a current of 40 mA were employed. The data were collected using a continuous scan mode with an angular speed of 2°/min for the angular range 5° ≤ 2θ ≤ 80°.

#### 2.3.3. Fourier Transformed Infrared Spectroscopy

The interactions between Ch and F were evaluated by Fourier transform spectroscopy (FTIR) (Thermo Scientific Nicolet iS50) in a frequency range of 400–4000 cm^−1^. Before the measurements, samples were dried at 24 °C, and they were mixed with dry KBr powder and compacted.

#### 2.3.4. Z Potential Measurements

Zeta potential (ζ) was acquired using a Malvern Zetasizer (NanoZs90). Samples were dispersed in distilled water and sonicated before the acquisition was performed.

### 2.4. Biological Studies

#### 2.4.1. Cell Culture

Vero cells derived from African green monkey kidney (ATCC^®^ CCL-81), HRT-18 (ATCC ^®^ CCL-244) from intestine adenocarcinoma, and A549 cells from human lung adenocarcinoma (ATCC^®^ CCL-185) were grown in Dulbecco Eagle’s medium (D-MEM, Gibco™ Thermo Fisher Scientific, Waltham, MA, USA) supplemented with 5% fetal bovine serum (FBS, Gibco™ Thermo Fisher Scientific, Waltham, MA, USA), 0.5% gentamicin (Sigma-Aldrich, St. Louis, MO, USA), and 1.5 g/L of sodium bicarbonate. Cell cultures were incubated at 37 °C and maintained in a humid atmosphere of 5% CO_2_. Cells were seeded at a density at which after 24 h of incubation they were 90% confluent.

#### 2.4.2. Viruses Stock

The experiments were performed using several viruses (see Table 1): human herpes simplex type 1 (HSV-1) strain Kos, HSV-1 Tk (acyclovir-resistant B2006), HSV-2 strain G, human respiratory syncytial virus (RSV) strain A2, human adenovirus (ADV) type 5, bovine coronavirus (BCoV) strain Mebus, and human polio virus type 1 (PV-1). Viral stocks of HSV, RSV, and PV-1 were propagated and quantified in Vero cells. Viral stock preparation and titration assays of BCoV were performed in human rectal adenocarcinoma cells (HRT-18) in a culture medium without fetal bovine serum and with 0.05% trypsin. A549 cells were used for ADV stock. Aliquoted viral stocks were stored at −80 °C. For this purpose, cells were infected and incubated at 37 °C in a 5% CO_2_ atmosphere, for 48–72 h post infection (p.i.) until the monolayer presented a predominant cytopathic effect (CPE) upon using an inverted microscope. The samples were lysed by two freeze–thaw cycles and stored at −80 °C. Viral titers were determined by the PFU (plaque-forming units) technique [36].

#### 2.4.3. Cytotoxicity Assay

Cell viability was measured by the 3-(4,5-dimethylthiazol-2-yl)-2,5-diphenyl tetrazolium bromide (MTT, Sigma-Aldrich, St. Louis, MO, USA) method. Serial two-fold dilutions of the lyophilizates were performed four times, starting from a maximum concentration of 10 mg/mL. Confluent cell cultures in 96-well plates were exposed to different concentrations of F or Ch, using incubation conditions equivalent to those used in antiviral assays. After 72 h of incubation, the supernatant was removed, and 0.1 mL of 5 mg/mL MTT/D-MEM reagent was added to each well. After 2 h incubation at 37 °C, the supernatants were removed, 100 µL of ethanol was added per well to solubilize the formazan crystals, and absorbance was measured at 595 nm. Wells containing only ethanol were used as blanks and subtracted as background for each sample. The results were expressed as a percentage of cell viability in relation to the untreated cells, considering this value to be 100%.

#### 2.4.4. Antiviral Assay

The cell cultures were infected with 50–100 PFU/well in the presence or absence of F (10–1000 µg/mL) or Ch (15–6000 µg/mL). Cultures were incubated for 1 h at 37 °C, with shaking every 15 min. The inoculum was then discarded, and a mixture of MEM 3% FBS and 1.4% methylcellulose (plating medium) was added in equal parts. The infected culture was incubated at 37 °C for 2–3 days in a controlled humid atmosphere with 5% CO_2_. Cells were fixed with 10% formalin for 20 min and washed and stained with 1% crystal violet. Viral infectivity was quantified by the PFU technique. To determine the percentage of PFU reduction, the following formula was used: % reduction = 100 − [(average viral plaques in the control condition − average viral plaques in the treated condition)/(average viral plaques in the control condition)] ×100.

#### 2.4.5. Mechanism of Antiviral Action Against HSV-1

Doses of 100 µg/mL of F or 300 µg/mL of Ch were administered for each treatment. The viral replication mechanism was tested according to the method of Mandal [37], with slight modifications.

For the following studies, cells were infected with approximately 50–100 PFU/well under different treatment conditions, as indicated below.

For viral adsorption, cells were infected in the presence or absence of F or Ch for 1 h at 4 °C. Then, the inoculum was removed, cells were washed with cold phosphate-buffered saline (PBS), and plating medium was added.

For viral internalization, cells were infected in the absence of F or Ch for 1 h at 4 °C. Cells were then washed twice with cold PBS and incubated for 1 h at 37 °C in D-MEM with or without F or Ch. Following this, cells were washed with cold PBS, and 0.1 mL citric buffer (40 mM citric acid, 10 mM KCl, 135 mM NaCl, pH 3.0) was added for 1 min to remove the adsorbed virus that had not been internalized. Cells were again washed with PBS and incubated with plating medium in the absence of F or Ch.

Post-adsorption, cells were infected in the absence of F or Ch for 1 h at 4 °C. Unadsorbed virus was then removed, and cells were washed twice with cold PBS and further incubated for 3 h at 37 °C. Finally, the cells were incubated with a plating medium in the presence or absence of F or Ch.

Cells were consistently infected in the presence of F or Ch for 1 h at 4 °C. After adsorption, unadsorbed virus was then removed and cells were washed twice with cold PBS and then incubated with plating medium with or without F or Ch.

For all treatments, PFUs were counted after two days of incubation at 37 °C, and results were expressed as inhibition (%) for each treatment with respect to the untreated infected cell control.

#### 2.4.6. Virucidal Activity

To determine, the virucidal activity of F or Ch, 20 µL of a viral suspension containing 10^3^ PFUs was exposed to F (10–1000 µg/mL) or Ch (15–6000 µg/mL) for 30 min at room temperature, and then the residual infectivity was titrated by the PFU technique [33]. A viral inoculum treated with medium without F or Ch was used as a control. Two experiments for each virus were performed, each in duplicate.

To determine the virucidal activity of biocomposites, we used 20 µL of a viral suspension containing 10^3^ PFU with the same volume of the corresponding F:Ch biocomposites. The final concentrations for Ch and F in the biopolymers were 428 µg/mL of F and 428 µg/mL of Ch in 1:1; 214 µg/mL of F and 428 µg/mL of Ch in 1:2; and 856 µg/mL of F and 428 µg/mL of Ch in 2:1 biocomposites. After 10 min, twice the volume of NaCl (2M) was added to desorb the virion to the F:Ch biocomposites, as detailed in previous reports [20,21]. For viral controls, the same procedure was used as for the treatments by mixing viral suspension with the D-MEM.

For F, Ch or biocomposite treatments, the mix was then diluted in cold D-MEM to determine residual infectivity by PFU, as described above, using Vero or A549 cells according to the virus tested. The dilution of the mix was effective in reducing the drug used by more than 100 times so that the viral titers were only due to the free virion.

The plates were then incubated at 37 °C with CO_2_ for the time specified for each virus (see Table 1), depending on the duration of each viral replication cycle.

#### 2.4.7. Transmission Electron Microscopy

Firstly, 20 µL of HSV-1 Kos containing 10^3^ PFU was exposed to 20 µL of formulation for 10 min. Then, the viruses were stained with uranyl acetate, and their viral morphology was examined by transmission electron microscopy (TEM) using a JEOL 100 CX II (JEOL, Tokyo, Japan 1983) microscope.

#### 2.4.8. Antioxidant Properties: Determination of Scavenger Activity

Scavenger activity was estimated based on the discoloration of a 2,2-diphenyl-1-picrylhydrazylradical (DPPH) solution, following the procedure as described by Montiel [38] with slight modifications. A 73 µM solution of DPPH in ethanol–distilled water (1:1) was prepared and measured by UV–visible spectrophotometry at 517 nm immediately (Absi). Then, different concentrations of compounds (F, Ch, biocomposites or vehicle) were added to 1 mL of DPPH solution and incubated for 30 min in the dark. Subsequently, the absorbance at 517 nm (Absf) was measured, and the percentage of antioxidant activity was calculated as follows:

$$\%\;DPPH\;scavenging\;=\;\left(\frac{Absi-Absf}{Absi}\right)\;\times \;100$$

### 2.5. Statistical Analysis

GraphPad Prism version 7 software (San Diego, CA, USA) was used to perform the corresponding statistical analyses. The results were compared using ANOVA followed by Tukey’s test, with *p* < 0.05 considered significant. Results represent the average of at least three experiments with each condition ± SD (standard deviation) unless otherwise noted.

## 3. Results

### 3.1. Synthesis and Characterization of Biomaterials

**Figure 1:**
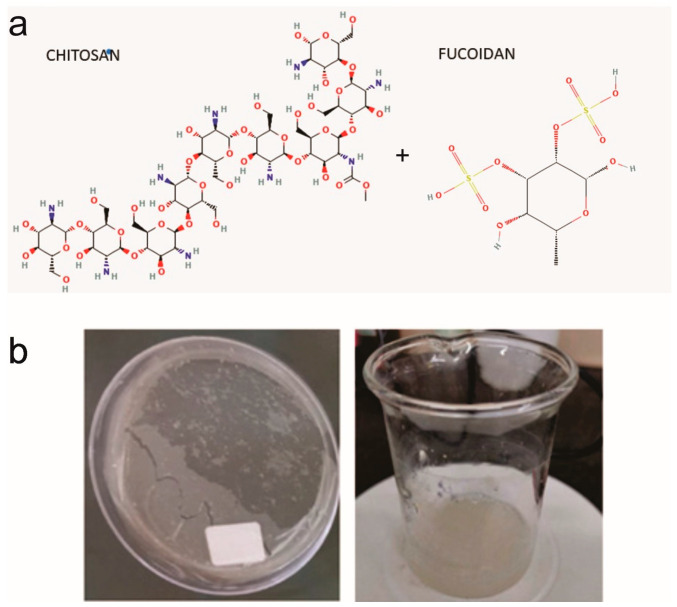
(**a**) Chemical structure of biopolymers. (**b**) Images of the obtained biomaterials as an aqueous dispersion and film biocomposites.

The preparation methodology induced the electrostatic interaction between the sulfate groups of F and protonated amine groups of Ch. Hence, the synthetic procedure was designed at pH 6, an intermediate value, to promote electrostatic and hydrogen bond interactions between the groups of both polyelectrolytes, as illustrated in Figure 1. This figure also shows the appearance of the resulting biomaterials, highlighting their excellent film-forming ability.

The fucoidan obtained (F) showed the presence of important proportions of neutral carbohydrates (51.9 ± 1.9%) and sulphate ester groups (17.9 ± 0.6%). Fucose and galactose were the main monosaccharides (43 ± 0.98 and 49 ± 0.84 galactose mol%, respectively), with minor proportions of rhamnose (1 mol%), arabinose (3 mol%), glucose (2 mol%), and mannose (2 mol%) and traces of xylose.

**Figure 2:**
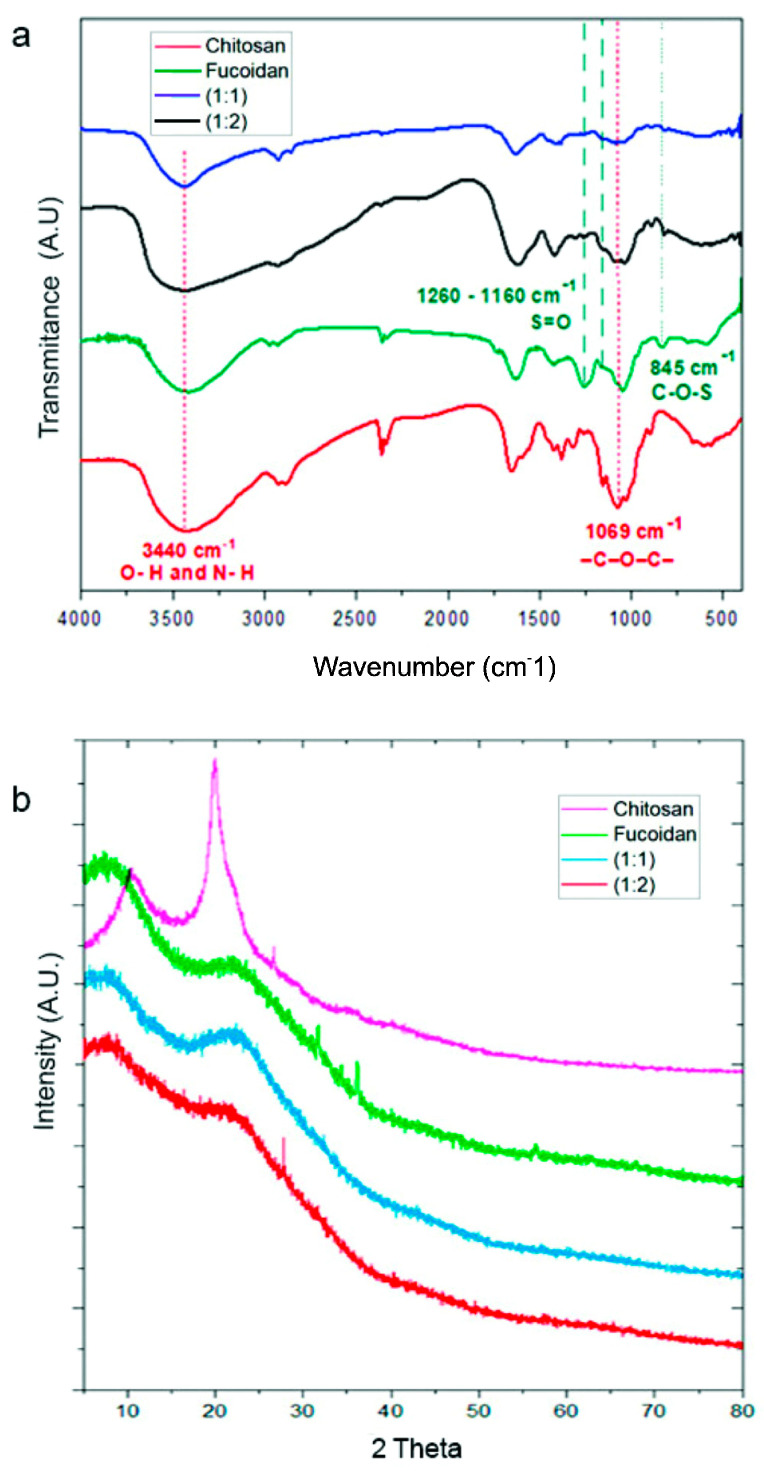
(**a**) FTIR spectra of biomaterials and raw polymers. (**b**) X-ray diffractogram of biomaterials and raw polymers. Arbitrary units (A.U.).

Formation of the biomaterials was verified by FTIR spectroscopy. Figure 2a shows the spectra of the resulting products and the raw biopolymers.

Characteristic Ch peaks distinguishable at 3440 cm^−1^ could be attributed to the vibration of OH and amine NH groups. The signals at 1370 and 1309 cm^−1^ could be assigned to –CH bending vibrations, while the peak at 1069 cm^−1^ corresponded to the skeletal frequency of –C–O–C– [39]. Additionally, a strong absorption peak at 1740 cm^−1^ was observed, attributed to the presence of carboxyl groups. The F spectrum showed distinctive signals at 1160–1260 cm^−1^ and 845 cm^−1^, associated with the vibration of S=O and C-O-S groups, respectively. However, some signals at 3300 cm^−1^ and 2800–2900 cm^−1^ overlapped with the Ch bands and were associated with OH and CH_2_-CH_3_, respectively.

It is worth mentioning that the formulation of F:Ch at a ratio of 2:1 was not characterized because the formation of fungus was evident during its storage even at 4 °C. Since the biocomposites were not added with any preservative, we considered this biomaterial insufficient to meet stability requirements for employment in the design of biomedical biocomposites.

The obtained values of ζ were 53.0, 46.6, and 10.3 mV for (F:Ch): 1:1, 1:2, and 2:1, respectively. They revealed aB reduction in the magnitude of the ζ as the proportion of F in biomaterials increased.

The diffractograms of biomaterials are depicted in Figure 2b, taking the one corresponding to raw biopolymers as a reference. The Ch diffractogram has previously been well documented in our own publications [20,21] and displayed two main diffraction peaks at around 2θ = 19° and 23°, which are associated with the hydrated conformation of Ch. The F diffractogram showed a slight signal at 2θ = 8° and 18°, with the latter partially overlapping the Ch peak, showing a most accentuated amorphous structure. Diffractograms corresponding to both biomaterials exhibited the same peaks as the biopolymeric precursors, with variations in intensity depending on the composition. This is consistent with the results obtained from the FTIR and Z potential data.

### 3.2. Antiviral Studies

#### 3.2.1. Antiviral Activity of F and Ch

**Figure 3:**
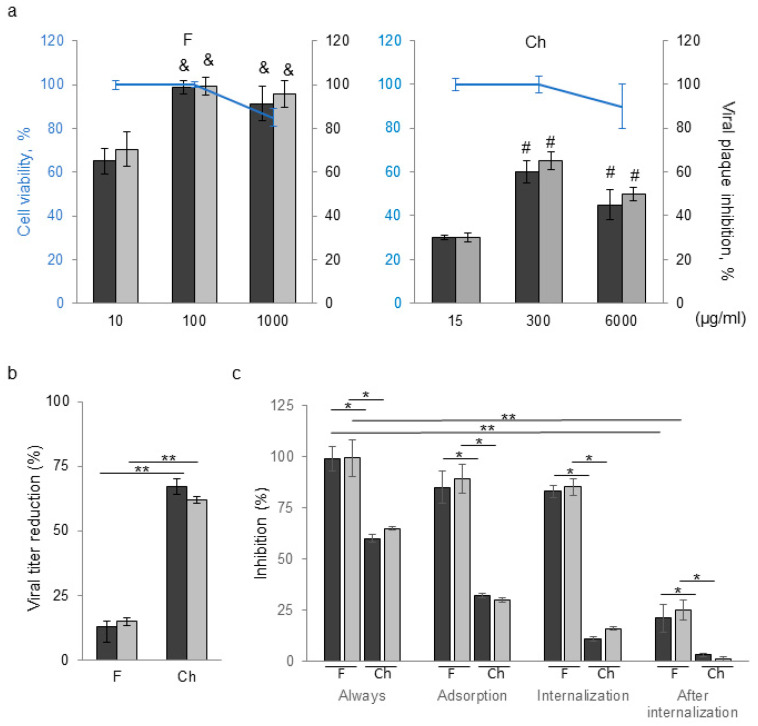
(**a**) Cell viability dose–response curve (blue lines) and antiviral assay for fucoidan (F) and chitosan (Ch) against BCoV (black bars) or HSV-1 (grey bars). (**b**) Virucidal assay of F (100 µg/mL) or Ch (300 µg/mL). (**c**) Cells infected under different treatment conditions in the presence of F (100 µg/mL) or Ch (300 µg/mL). Results are expressed as the mean ± SD of data from at least three separate experiments; each condition was processed in duplicate. * *p* < 0.05, ** *p* < 0.01, ^&^ *p* < 0.05 respect to to 10 µg/mL of F, ^#^ *p* < 0.05 with respect to 15 µg/mL of Ch.

In an initial series of experiments, we investigated the anti-BCoV and anti HSV-1 activity of F and Ch separately. Figure 3a illustrates that F exhibited antiviral properties close to 100% at 100 µg/mL for both viruses, without inducing cytotoxic effects. On the other hand, Ch demonstrated around a 65% antiviral effect for BCoV and HSV-1, without cytotoxic effects, at an extremely low concentration of 300 µg/mL. Additionally, we observed that the antiviral effect of F was mainly exerted by inhibiting virus adsorption and internalization into the cell, and we observed limited antiviral activity during the post-adsorption phase (Figure 3c) with a negligible virucidal effect (Figure 3b). Additionally, Ch exhibited its highest antiviral effect when applied as a pre-treatment of the virus before infecting the cell culture, indicating that its action was mainly virucidal (Figure 3b).

#### 3.2.2. Virucidal Activity of Biocomposites on HSV-1

The next objective was to evaluate the inactivating activity of the biocomposites developed with F and Ch. There is evidence that virion−fucoidan interaction cannot inactive virus permanently [40] so we focused on the study of the virucidal action of these biocomposites, aiming to disrupt the viral particle through avoiding any possible mechanism of viral infection. Therefore, we investigated whether the virucidal activity of fucoidan–chitosan biocomposites was improved over that of their original materials.

**Figure 4:**
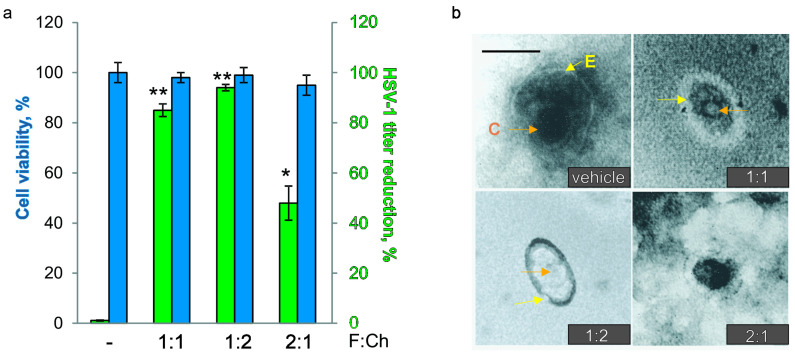
(**a**) HSV-1 Kos titer reduction in a virucidal (green) and cell viability (blue) assay. Means and SD are shown from three separate experiments. Each condition was processed in duplicate. * *p* < 0.05, ** *p* < 0.01 vs. vehicle. (**b**) Representative TEM micrographs of HSV-1 Kos exposed to biocomposites. Orange arrows indicate the viral capsid, and yellow arrows indicate the viral envelope of HSV-1 exposed to formulations. Scale bar: 200 nm.

Virucidal agents act directly on the viral particle, producing irreversible loss of infectivity, thereby hindering multiplication of the virus in host cells; they can destroy the virion structure (viral envelope, capsid, or genome) [41]. In this way, we aimed to obtain non-cytotoxic biocomposites that would enhance the virucidal action compared to F or Ch alone, managing to develop three F:Ch biocomposites (1:1; 1:2; 2:1). In this section, we performed the experiment with an enveloped virus, HSV-1, whose incubation time is shorter than the other enveloped viruses, such as BCoV or RSV (Table 1). In Figure 4a, it can be observed that the virucidal assay resulted in HSV-1 inactivation of <85% for the 1:1 biocomposites and of almost 95% for the 1:2 biocomposites, with both surpassing the virucidal effects of F and Ch alone (Figure 3c). However, the 2:1 formulation exhibited a lower virucidal effect (~50%) than 1:1 or 1:2, similar to Ch alone yet higher than F alone (Figure 3c). After viral pre-treatment, the HSV-1 structure was observed via TEM. In Figure 4b, TEM images show the intact structure of the untreated virus. In the case of the 1:1 or 1:2 biocomposites, the viral structure appears altered, with both the capsid (orange arrows) and the viral envelope (yellow arrows) undergoing changes after treatment. However, for the 2:1 formulation, the viral envelope and capsid are even more preserved.

#### 3.2.3. Virucidal Activity of Biocomposites on Several Viruses

**Figure 5:**
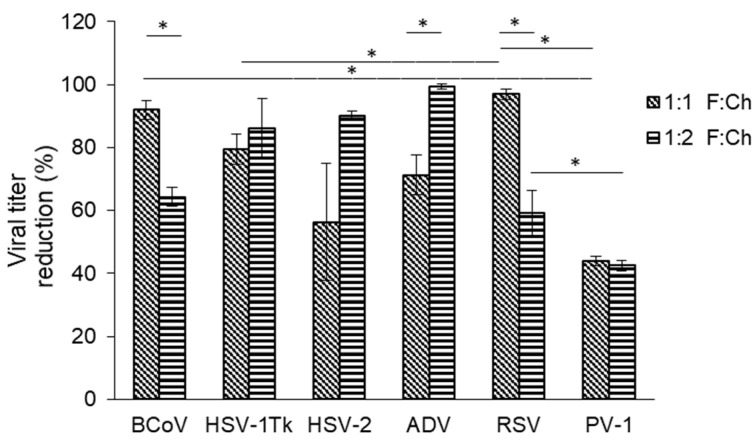
Virucidal activity of F:Ch biocomposites (1:1 or 1:2). Results are expressed as the mean ± SD of data from at least three separate experiments. Each condition was processed in duplicate. * *p* < 0.05 vs. each indicated treatment.

Since the F:Ch 2:1 formulation did not meet the stability requirements for use in the design of biomedical biocomposites (Figure 2a), and considering the superior virucidal activity demonstrated by the 1:1 and 1:2 biocomposites (Figure 4), we decided to investigate the spectrum of action of the 1:1 and 1:2 biocomposites on a range of different viruses. Among the viruses used in this study, RSV is known to be the one of the most labile, while poliovirus is one of the most resistant viruses existing in nature and that infects humans. Based on the general behavior of viruses and according to our results, we observed that the 1:1 formulation exhibited the highest antiviral effect against enveloped viruses with RNA genomes (such as RSV and BCoV) (~99%) and a comparatively lower effect on the more resistant non-enveloped PV-1. The 1:1 biocomposite showed intermediate action on the other viruses studied (Figure 5). In contrast, the 1:2 formulation, which demonstrated greater virucidal efficacy (Figure 4), had a more pronounced inactivating effect on DNA genome viruses, such as enveloped herpesvirus, ~85%, and non-enveloped viruses, such as ADV, ~100%. Interestingly, viruses with RNA genomes proved to be more resistant to the 1:2 biocomposites.

### 3.3. Scavenger Activity

**Figure 6:**
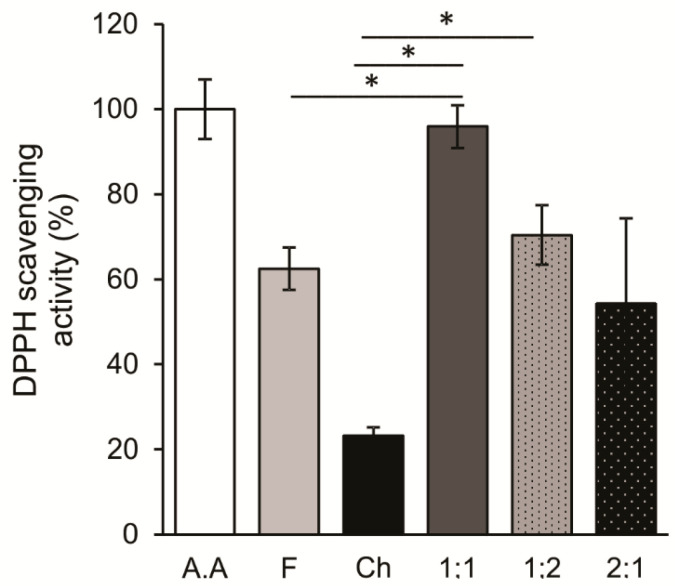
Scavenging effects on DPPH radicals by F, Ch, F:Ch biocomposites (1:1; 1:2; 2:1) and AA (ascorbic acid). Values represent the mean from three independent tests. * *p* < 0.05 vs. each indicated treatment.

Free radicals are highly toxic molecules for cells and can be produced during various conditions such as viral infections [42,43,44], exposure to UV radiation, lipid peroxidation, etc. [45]; their accumulation is associated with cellular aging [46]. Interestingly, several fucoidans have been reported to have antioxidant properties [47]. The DPPH assay is the commonly used method to investigate the antioxidant properties of these polysaccharides, using DPPH as the source of free radicals. In this study, we evaluated and compared the antioxidant activities of F, Ch, and F:Ch biocomposites, using ascorbic acid (AA) as a reference. From Figure 6, it can be seen that scavenging activity increased up to ~60% and 20% for F and Ch respectively. Notably, the scavenging activity was significantly higher in the F:Ch 1:1 formulation than the other biocomposites, and this activity was also comparable to the effects of AA.

## 4. Discussion

Even though the combination of chitosan and fucoidan can result in formulations with improved properties in terms of bioactivity and physicochemical properties, articles in the existing literature are scarce, and most of them are devoted to nanoparticulated drug delivery systems that can be orally or intravenously administrated [48,49]. In such cases, other therapeutic agents are added in order to confer the biomedical action [50]. In such articles, the antiviral properties are missing.

In this work, both biopolymers are employed simultaneously as the carrier and active ingredient, taking advantage of the antiviral properties of both biopolymers, promoting additional functionality (such as antioxidant properties), and proposing potential formulations for topcal administration to the skin or mucous membranes. The predominance of fucose and galactose in *Undaria pinnatifida* fucoidans has been reported by other authors as responsible for their antiviral behavior [51]. It is well known that fucoidans exhibit low bioavailability, and this condition may be attributed to seveal factors. Fractions of this polysaccharide are normally extracted from algae of diverse origins that may contain a mixture of other polysaccharides of different molecular weights, and crude extracts may contain large amounts of non-fucoidan components [52]. As already detailed, fucoidan contains a backbone of sulfated fucans, and its structure depends on the species of algae and harvest conditions [52]. These factors may alter the F bioavailability, especially when oral and topical administration are considered [53,54].

Combination with Ch was achieved by following a simple, low-cost, and easily scalable process. In this regard, it is worth noting that fucoidan is a strong polyelectrolyte with a pKa value between pH 2.5 and 3 depending on the F origin, whereas the pKa of amino groups of chitosan is around pH 6.2–6.5 [55]. Hence, contact between solutions from both biopolymers was induced near pH 6, aiming to induce strong electrostatic interactions favoring the formation of a polyelectrolyte complex (PEC) [56]. Given the chemical structure of F and Ch (Figure 1), possible interactions between them can be assumed.

Even though the existing literature concerning biomaterials composed of Ch and F is limited, a few articles have been published in recent years, supporting these ideas about biopolymers’ interactions. For instance, Carvalho et al. revealed that the sulfonated groups of F are responsive for the strong and stable interactions between biopolymers. For this reason, it was hypothesized that the biomaterials containing fucoidan in their formulations would have a more stable and cohesive structure associated with higher ionic bonds established with the positively charged amine groups from chitosan [57]. Interestingly, while previous studies have explored nanoparticle formation, this work proposes the development of adhesive patches for dermal application, offering a novel approach to utilizing these biomaterials. This filmogenic capability is provided by Ch moieties, since F is not a gel or film-forming biopolymer by itself (see Figure 1) [55].

The ζ data support the idea of the electrostatic nature of F and Ch interactions. Therefore, a partial neutralization of the positive Ch charge was evidenced when raising the proportion of F, leading to values of about 53.0, 46.6, and 10.3 mV for (F:Ch) in ratios of 1:1, 1:2 and 2:1, respectively, as measured in distilled water at pH 5–5.5. It is important to highlight that the achieved values for raw F and Ch were −40 mV and +60 mV, respectively [58].

The F:Ch 1:1 ratio seems to ensure the most efficient interactions between available functional groups of both biopolymers, not only from the chemical point of view but also when considering bioactive properties, as will be detailed later. Similar results were attained by Huang et al., who explored chitosan–fucoidan weight ratios of 1:1, 1:5, and 1:10 (and charge ratios of 1.66:1, 1.33:1, and 1.16:1) when designing nanoparticulated systems as drug delivery tools. They found nanoparticle (NP) sizes of 170, 280, and 250 nm and ζ-potentials of +25, −52, and −53 mV, respectively. A ratio of 1:1 was assumed to be the most suitable according to the NP properties [59].

Spectroscopic analysis developed by FTIR demonstrates the coexistence of both biopolymers through the presence of typical signals ascribed to the vibration of bonds from the main functional groups; this means OH and NH_2_ in the case of Ch and COO- and SOO in the case of F. The intensity of these signals is aligned with the nominal composition of the biomaterials in terms of the proportion of F:Ch. Similar results have been reported by other authors, who determined a noticeable shift in the C=O group which might be caused by a different environment around this group [48,49]. Significant evidence that the electrostatic interactions involve SOO and NH-OH groups has been provided by the absence of the band ascribed to the S=O group located at 1160–1260 cm^−1^. This signal is observable in the spectrum of raw F, but it is not detected in the formulations involving 1:1 and 1:2 F:Ch ratios.

Regarding the antiviral activity of fucoidans, it is known that these types of polymers can inhibit the entry of influenza, bovine viral diarrhea virus, human cytomegalovirus, HIV, and HSV-1 by preventing viral adsorption onto cells. Fucoidan inhibits the virus’s ability to adhere to host cells by interacting with the positively charged region of viral envelope glycoproteins that are crucial for attachment [40]. Consistent with this, we observed that the antiviral effect of F was mainly exerted by inhibition of virus adsorption and internalization into the cell, and there was limited antiviral activity during the post-adsorption phase (Figure 3c) with a negligible virucidal effect (Figure 3b). On the other hand, the precise antiviral mechanism of chitosan and its derivatives is still unclear, despite the large amount of published research. The direct destruction of the virus, the disruption of the virus’s protective envelope by electrostatic interaction between the polycationic positive charge of chitosan and its negatively charged surface, the inhibition of viral adsorption and subsequent host cell invasion, and other mechanisms are some of the antiviral mechanisms that have been proposed for chitosan [60]. In this work, our results showed that Ch exhibited its greatest antiviral effect when applied as a pre-treatment of the virus before infecting the cell culture, indicating that its action was mainly virucidal and is limited, as reported for Ch in other studies [20,21].

Next, we set out to create non-cytotoxic biocompounds with a greater antioxidant effect and virucidal activity than their basic components. Virucidal agents act directly on the viral particle, producing irreversible loss of infectivity, thereby hindering the multiplication of the virus in the host cells; they can destroy the virion structure, viral envelope, capsid, or genome [41]. Based on other works, the antiviral behavior of F:Ch 2:1 biocomposites suggests a virustatic effect on enveloped virus HSV-1, since the integrity of the virion structure was not lost, while 1:1 or 1:2 biocomposites are associated with a virucidal effect, as reflected by the loss of structural integrity in virions [45]. In general, the most accepted virucidal mechanisms for cationic compounds have been associated with the direct binding of antimicrobial compounds to viral particles [61]. The charge of virucidal compounds and viruses plays an indispensable role. Virucidal compounds, which are positively charged compounds, can interact with viruses that are negatively charged by electrostatic interactions which alter viral capsid structure and viral nucleic acids [61]. However, electrostatic interactions alone are insufficient to create a persistent binding and virucidal activity. It has been postulated that to transform a compound into a virucidal compound, there must be a balance between electrostatic interactions and its hydrophobicity. The key for the change in mechanism to irreversible inhibition (virucidal) is the addition of multivalent hydrophobic interactions to the established electrostatic ones. The combination of electrostatics with hydrophobic interactions can be exploited to make broad-spectrum antivirals [62]. Chitosan is a positively charged compound. This compuond also has hydrophilic (amine and hydroxyl groups) and hydrophobic (acetyl groups) sites, but the number of hydrophobic sites is relatively small. It has been shown that the antiviral activity of sulfated polysaccharides is reversed by dilution, but modification of these molecules with hydrophobic groups transforms them into virucidal materials [61]. In studies carried out in this work, we observed that chitosan has higher virucidal activity than F; in addition, the 1:2 compound had greater virucidal action than 1:1, 2:1, and Ch or F indiviually. A synergism was observed in the virucidal action of 1:2 as compared to F and Ch alone. A possible explanation could be that the electrostatic interactions between SOO and NH-OH in the F:Ch formulations could slightly alter the arrangement of hydrophobic sites in the formulations. These data suggest that in the formulations, the rearrangements produced by the F interactions with Ch could promote the interaction with the negatively charged viruses, favoring the destruction of the virions, possibly through the new balance between electrostatic interactions and hydrophobicity.

Our results demonstrate that enveloped viruses with RNA genomes are more resistant to the virucidal activity of 1:2 biocomposites. The difference in antiviral activity on the viruses could be related to their capsid structure; type of genome; nucleocapsid stability against antiviral effects; virus size, tendency toward viral aggregation; the presence or lack of a viral envelope; the mode of action of the biocomposites; and the resistance mechanisms of viruses (which have rarely been documented) [62]. These results suggest that the antiviral effect depends both on the virus and the chemical structure of the antiviral formulation. This observation provides valuable insights into the differential mechanisms of action of 1:1 and 1:2 biocomposites across various viruses.

It is known that polysaccharides’ biological activities depend on their molecular structure [40]. The antioxidant properties of fucoidans are primarily attributed to their degree and distribution of sulphate groups [47], which shows that the conformation of the main chain and the distribution of sulphate groups in it could alter the antioxidant activity of a polymer. Our results suggest that the interactions between F and Ch may induce conformational changes in the structures of both components, leading to a different arrangement of the sulphate groups, as observed in Figure 1a. This altered arrangement may be more favorable for scavenger activity, providing insights into the potential synergistic antioxidant effects of F:Ch biocomposites [63].

## 5. Conclusions

The successful production of biocomposites based on natural polysaccharides was achieved. Their characterization showed a strong dependence between surface properties and the composition of biocomposites. The studies performed show that the 1:1 and 1:2 biocomposites of F:Ch possess antiviral and scavenger activity; however, the 2:1 formulation did not demonstrate suitable antiviral or stability characteristics. Furthermore, 1:1 and 1:2 biocomposites created using non-cytotoxic composites showed broad-spectrum antiviral activity, even against coronavirus. The primary mechanism of action was virucidal activity, leading to the alteration of viral particle integrity. The action of biocomposites against these viruses was found to be fast, with an effect observed after 10 min of exposure. In view of these promising results, these biocomposites could be considered interesting tools for the topical administration of treatments for mucosal infections, skin diseases associated with different viral infections, current viruses, and emerging viruses.

## Figures and Tables

**Table 1:** Characteristics of the viruses employed.

Virus	Envelope	Genome	Incubation Times (Days)
HSV-1	Enveloped	DNA	2
HSV-1 Tk	Enveloped	DNA	2
HSV-2	Enveloped	DNA	2
RSV	Enveloped	RNA	5
BCoV	Enveloped	RNA	3
ADV	Non-enveloped	DNA	7
PV-1	Non-enveloped	RNA	2

## Data Availability

No new data were created. Data are unavailable due to privacy or ethical restrictions; a statement is still required.
